# Olive (*Olea europaea* L.) Leaf Polyphenols Improve Insulin Sensitivity in Middle-Aged Overweight Men: A Randomized, Placebo-Controlled, Crossover Trial

**DOI:** 10.1371/journal.pone.0057622

**Published:** 2013-03-13

**Authors:** Martin de Bock, José G. B. Derraik, Christine M. Brennan, Janene B. Biggs, Philip E. Morgan, Steven C. Hodgkinson, Paul L. Hofman, Wayne S. Cutfield

**Affiliations:** 1 Liggins Institute, University of Auckland, Auckland, New Zealand; 2 Heart Research Institute, University of Sydney, Sydney, Australia; 3 Gravida: National Centre for Growth and Development, Auckland, New Zealand; College of Tropical Agriculture and Human Resources, University of Hawaii, United States of America

## Abstract

**Background:**

Olive plant leaves (*Olea europaea* L.) have been used for centuries in folk medicine to treat diabetes, but there are very limited data examining the effects of olive polyphenols on glucose homeostasis in humans.

**Objective:**

To assess the effects of supplementation with olive leaf polyphenols (51.1 mg oleuropein, 9.7 mg hydroxytyrosol per day) on insulin action and cardiovascular risk factors in middle-aged overweight men.

**Design:**

Randomized, double-blinded, placebo-controlled, crossover trial in New Zealand. 46 participants (aged 46.4±5.5 years and BMI 28.0±2.0 kg/m^2^) were randomized to receive capsules with olive leaf extract (OLE) or placebo for 12 weeks, crossing over to other treatment after a 6-week washout. Primary outcome was insulin sensitivity (Matsuda method). Secondary outcomes included glucose and insulin profiles, cytokines, lipid profile, body composition, 24-hour ambulatory blood pressure, and carotid intima-media thickness.

**Results:**

Treatment evaluations were based on the intention-to-treat principle. All participants took >96% of prescribed capsules. OLE supplementation was associated with a 15% improvement in insulin sensitivity (p = 0.024) compared to placebo. There was also a 28% improvement in pancreatic β-cell responsiveness (p = 0.013). OLE supplementation also led to increased fasting interleukin-6 (p = 0.014), IGFBP-1 (p = 0.024), and IGFBP-2 (p = 0.015) concentrations. There were however, no effects on interleukin-8, TNF-α, ultra-sensitive CRP, lipid profile, ambulatory blood pressure, body composition, carotid intima-media thickness, or liver function.

**Conclusions:**

Supplementation with olive leaf polyphenols for 12 weeks significantly improved insulin sensitivity and pancreatic β-cell secretory capacity in overweight middle-aged men at risk of developing the metabolic syndrome.

**Trial Registration:**

Australian New Zealand Clinical Trials Registry #336317.

## Introduction

It is estimated that 20–50% of the European population use complementary or alternative therapy to treat disease or to help prevent its onset [1]. In Britain, approximately 40% of general practitioners provide complementary therapies for their patients [2]. With respect to type 2 diabetes, one third of patients actively use alternative medicine to manage their disease, despite the paucity of scientific evidence to support its use [3]. The leaves of the olive plant (*Olea europaea* L.) have been used for centuries in folk medicine to treat diabetes [4]. Recently, the medicinal properties of olive products have focussed on its polyphenols (particularly oleuropein and hydroxytyrosol), which according to animal and *in vitro* studies have antioxidant, hypoglycaemic, antihypertensive, antimicrobial, and anti-atherosclerotic properties [5]. Polyphenols are found in most edible plants, and are reportedly responsible for the health benefits associated with the consumption of chocolate, coffee, green tea, and red wine [6].

The nutraceutical market exploring the potential health benefits of olive products is expanding. The concentration of olive plant polyphenols is far greater in the leaves than in the fruit or fruit oil, and the leaves that were once discarded as by-products of tree pruning are now considered a valuable commodity. However, while the cardiovascular health benefits of a Mediterranean diet rich in olive oil is well established [7], clinical studies examining the effects of olive polyphenols supplementation on cardiovascular disease risk are scarce, flawed, or contradictory. Thus, although the European Food Safety Authority has endorsed the health claim that “the consumption of olive oil polyphenols contributes to the protection of blood lipids to oxidative damage”, it has rejected several other health claims [8].

There are very limited data examining the effects of olive polyphenols on glucose homeostasis in humans. Thus, we conducted a randomized, double-blinded, placebo-controlled, crossover trial to assess whether supplementation with olive leaf polyphenols would affect modifiable cardiovascular risk factors in overweight males, who by virtue of their body mass are likely to be insulin resistant. In addition, plasma markers involved in the development of cardiovascular disease were investigated. Potential mechanisms underpinning the clinical outcomes were also examined.

## Methods

### Ethics Statement

Ethics approval for this study was provided by the Northern Y Regional Ethics Committee (New Zealand Ministry of Health), and written informed consent was obtained from all participants. This study was registered with the Australian New Zealand Clinical Trials Registry (#336317). The protocol for this trial and supporting CONSORT checklist are available as supporting information (see Checklist S1 and Protocol S1).

### Subjects

Overweight males (BMI 25–30 kg/m^2^) aged 35–55 years were eligible to participate. Volunteers were recruited in February 2011 via advertisements in local newspapers that circulate freely in the central Auckland metropolitan area. Exclusion criteria were: illicit drug use (including tobacco), diabetes, or being on medications likely to affect insulin sensitivity. Subjects taking antihypertensive or lipid-lowering medications were recruited, but were required to have been on a stable dose for at least 6 months prior to start of the study. These subjects were also encouraged not to change dose throughout the trial, and doses were checked at each assessment. Further, all participants were asked not to make any substantial alterations to their lifestyle for the duration of the trial. Specifically, participants were instructed not to make changes to their diet and physical activity levels.

### Randomization and Masking

Randomized allocation was done using computer random number generation. The code was kept by an independent third party, and was not released until after statistical analysis. Both researchers and subjects were ‘blinded’ to the contents of capsules being taken. To maintain integrity of the trial evaluation, statistical analyses were carried out on encoded data, such that the analyst (JGBD) was also ‘blinded’ to treatment.

### Study Design

This was a 30-week randomized, double-blinded, placebo-controlled, crossover trial. Participants were randomized to receive capsules with olive leaf extract (OLE) or placebo (Comvita, Auckland, New Zealand) for 12 weeks, which is the minimum study period that can reliably detect a sustained effect of dietary intervention [9]. Participants then switched over to the other treatment after a 6-week washout period. The polyphenol content of the OLE was independently verified (Table 1). Participants were instructed to take four capsules as a single dose, once a day, with a glass of water, equating to a daily dose of 51.1 mg oleuropein and 9.7 mg hydroxytyrosol for participants on active treatment. OLE was suspended in safflower oil, while placebo capsules contained safflower oil only. Importantly, placebo and active capsules were both odourless and identical in appearance (opaque green soft capsules), size, and grade.

**Table 1:** Total polyphenol content of each daily dose of olive leaf extract.

Compound	Content in 4 capsules (mg)
Oleuropein	51.124
Hydroxytyrosol	9.666
Kaempferol	0.021
Apigenenin	0.046
Flavonoid	0.028
Verbascoside	0.344
Phenolic acids (calculated as caffeic acid)	0.233
Oleic acid	0.013
Quercetin	0.038
Luteolin	0.249
Rutin	0.150

**Figure 1:**
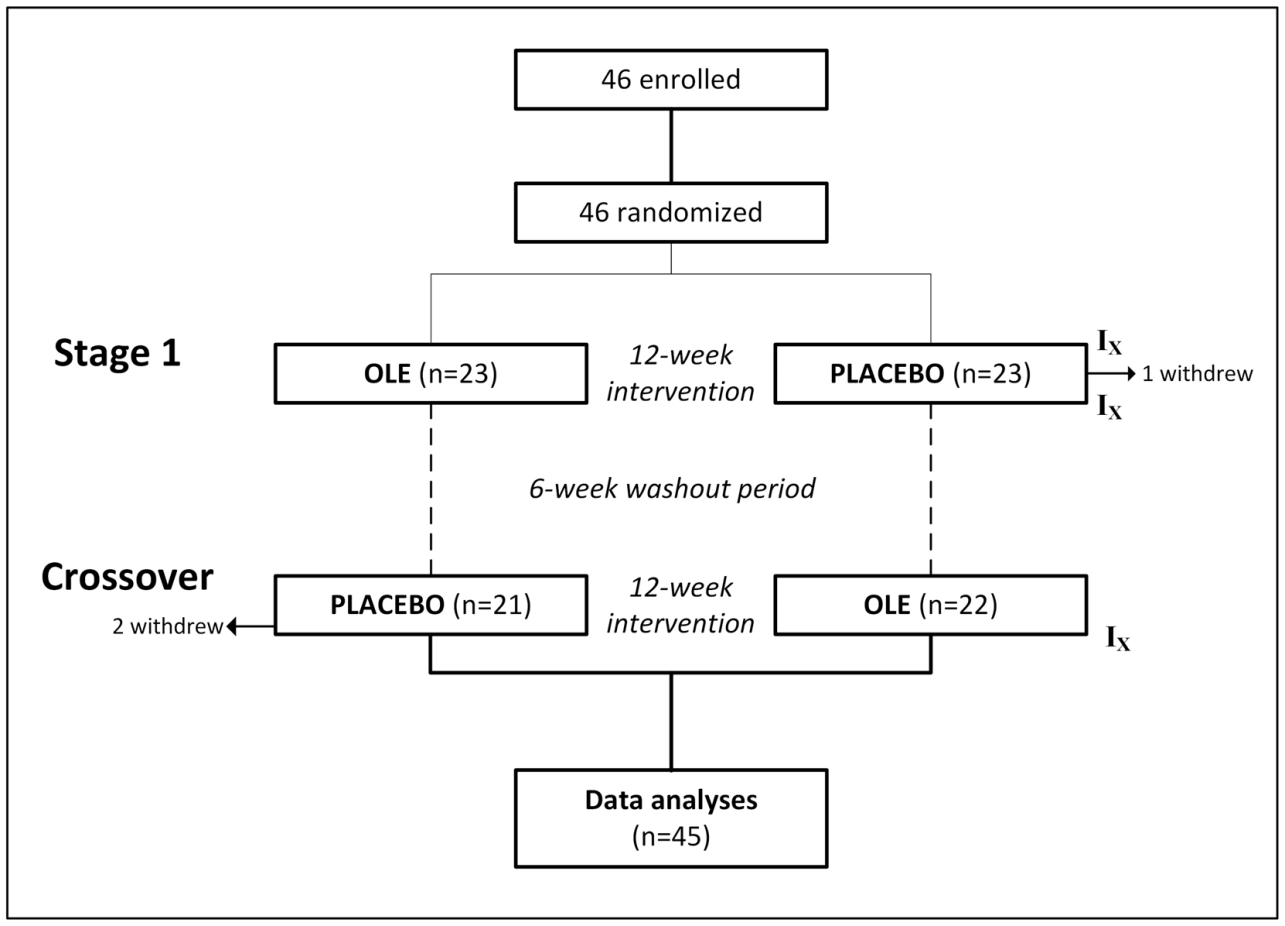
Summary of study’s recruitment process and trial execution. I_X_ indicates timing of assessments. One participant withdrew from the study during stage 1 due to injury, while the two subjects that withdrew after crossover were either lost to follow up or to the developing acne.

We have shown that following ingestion of an identical dose of OLE, olive polyphenol metabolites in plasma peak after 80 minutes and are cleared by 240 minutes (de Bock et al, unpublished data). Nonetheless, we chose a generous 6-week washout period, after which participants crossed to the opposite intervention (Figure 1). All clinical assessments were carried out between 06∶30 and 08∶30 at the Maurice & Agnes Paykel Clinical Research Unit (Liggins Institute, University of Auckland), after an overnight fast and no strenuous activity over the previous 24 hours. Participants were instructed not to take their assigned capsules on the morning of investigation. Subjects were assessed at the start of the study, and at the end of each intervention phase. Blood samples were collected and placed on ice; following separation, plasma and serum were stored at −20 and −80°C, respectively, for later analysis.

### Primary Outcome

The primary outcome was insulin sensitivity, assessed via a 75 g oral glucose tolerance test. Insulin sensitivity (ISI) was assessed using the Matsuda method, with glucose and insulin samples collected at 0, 30, 60, 90, and 120 minutes [10]. The Matsuda method has a strong correlation with the hyperinsulinemic euglycaemic clamp (r = 0.77) [11], and excellent reproducibility during multiple measures [12].

### Secondary Outcomes

Other parameters of glucose homeostasis assessed included pancreatic β-cell function, also calculated from the oral glucose tolerance test: the product of insulin sensitivity (derived by the Matsuda method) and the change in glucose and insulin over the first 30 minutes (oral disposition index) [13]. Glucose and insulin profiles after the glucose challenge were calculated and expressed as the area under the curve (AUC).

To identify potential underpinning mechanisms, fasting blood samples were used to assess cytokines known to influence glucose metabolism: insulin-like growth factor I (IGF-I), IGF-II, IGF binding protein 1 (IGFBP-1), IGFBP-2, IGFBP-3, ultra-sensitive C-reactive protein (CRP), tumor necrosis factor-alpha (TNF-α), interleukin-6, and interleukin-8.

Fasting blood samples were also used to assess lipid profile, namely triglycerides, total cholesterol, high-density lipoprotein cholesterol (HDL-C), and low-density lipoprotein cholesterol (LDL-C). Liver function tests were also performed at each assessment, with measurements of aspartate aminotransferase (AST), alanine aminotransferase (ALT), alkaline phosphatase (ALP), and gamma-glutamyl transferase (GGT).

Auxological assessment included height measurement using a Harpenden stadiometer. Weight and body composition were assessed using whole-body dual-energy X-ray absorptiometry (DEXA, Lunar Prodigy 2000, General Electric, Madison, USA). Body composition data of interest were total percentage body fat and the ratio of android fat to gynoid fat. Note that android and gynoid fat values were determined by the manufacturer’s software, based on an automated sectioning of specific areas of the body [14].

24-hour ambulatory blood pressure monitoring was carried out prior to each clinical assessment. Participants were fitted with a Spacelabs 90207 or 90217 (Spacelabs Medical Inc., Redmond, USA), with each subject being assigned the same device model for all assessments. Measurements were performed every 20 minutes between 07∶00 and 22∶00, and every 30 minutes from 22∶00 to 07∶00. Only profiles with a total of at least 40 readings over a 24-hour period were analysed [15].

Carotid intima-media thickness (cIMT) was also measured to assess possible treatment effects, as it is a validated and reproducible measure that is predictive of cardiovascular and cerebrovascular risks [16]. cIMT was measured using an M-Turbo ultrasound system (Sonosite, Bothel, USA) by a trained investigator [MdB], with images attained using a standard protocol [17]. The far wall of the right common carotid artery was used for all three assessment points. Digitally stored images were analysed by a single reader [MdB] using computer software automated callipers (SonoCalctm v.4.1, Sonosite). A maximal cIMT measurement approximately 10 mm proximal to the carotid bulb was used for comparative analysis. To assess reproducibility, triplicate measures were taken of seven healthy volunteers over a 7-day interval, and resulted in an intra-observer CV of 3.7% (unpublished data).

Lifestyle factors were recorded with an itemised food diary and a physical activity recall. Three-day dietary records were collected at baseline and at clinical assessment following each 12-week intervention. Each dietary report encompassed an itemized nutritional intake recorded during two week days (Monday to Friday) and one weekend day. Nutritional intake was recorded using standard household measures, as well as the information from food labels where appropriate. Participants were instructed by a trained investigator [MdB], who also reviewed all food records with each participant to address unclear descriptions, errors, omissions, or doubtful entries. Records were subsequently entered into Foodworks software (v6.0, Xyris Software, Brisbane, Australia) by the trained investigator [MdB]. Physical activity levels were assessed using the International Physical Activity Questionnaire (IPAQ) [18], covering four domains of physical activity: work-related, transportation, housework/gardening, and leisure time.

In addition, subjective measures of wellbeing were assessed by the Medical Outcomes Study Short Form (SF-36: New Zealand/Australia adaptation). The SF-36 is a validated tool that measures perception of health on eight multi-item dimensions covering functional status, wellbeing, and overall evaluation of health [19].

### Assays

Insulin concentrations were measured using an Abbott AxSYM system (Abbott Laboratories, Abbott Park, USA) by microparticle enzyme immunoassay with an inter-assay coefficient of variation (CV) of 5.4%. Glucose concentrations were measured on a Hitachi 902 autoanalyser (Hitachi High Technologies Corporation, Tokyo, Japan) by enzymatic colorimetric assay (Roche, Mannheim, Germany) with a CV of 2.1%. Commercially available ELISAs were used to measure plasma IGF-I, IGF-II, IGFBP-1, IGFBP-2, and IGFBP-3 (Meddiagnost, Reutlingen, Germany) with CV of 3.5, 0.9, 3.6, 8.8, and 8.5%, respectively). Commercially available ELISA kits were used to evaluate TNF-α, interleukin-6, and interleukin-8 (Invitrogen, Carlsbad, USA) with CV of 9.3%, 7.4, and 3.4%, respectively, and oxidised LDL-C (Mercodia, Uppsala, Sweden) with a CV of 5.7%. Commercially available ELISA kits were used to evaluate ultra-sensitive CRP (USCN Life Science, Wuhan, China) with a CV of 10%. Triglycerides, total cholesterol, HDL-C, LDL-C, AST, ALT, ALP, and GGT concentrations were measured on a Hitachi 902 autoanalyser (Hitachi High Technologies Corporation) by enzymatic colorimetric assay (Roche) with a CV lower than 2.5%.

### Sample Size

The power calculation was based upon a known mean adult Matsuda index of 15.6 and standard deviation of 8.7 [20]. A sample of 46 participants in total would have at least 80% power at 5% level of significance (two-sided) to detect a 25% difference in Matsuda index with and without OLE. This was based on the assumption of a correlation of 0.5 between measurements on the same subject, and a 10% drop out rate during the study.

### Statistical Analysis

Treatment evaluations (i.e. OLE vs placebo) were based on the principle of intention-to-treat (ITT). All statistical tests were two-sided and a 5% significance level maintained throughout the analyses. Statistical analyses were performed in SAS v.9.2 (SAS Institute, Cary, USA). Linear mixed models were used to assess the main treatment effect accounting for randomization sequences and time periods. Importantly, regression models also adjusted for the baseline value of the outcome response to gain statistical efficiency and power (i.e. baseline data were included in the model as covariates). Other confounders that were considered in the analysis included: on-going use of medication (for cholesterol or hypertension), IPAQ scores, age, and total body fat percentage (from DEXA scans). When necessary, response variables were log-transformed to approximate normality. Baseline descriptive data are presented as mean ± standard deviation (SD). The results from linear mixed models are expressed as model-adjusted means and 95% confidence intervals.

## Results

Forty-six eligible participants were randomized into the trial (Figure 1). Four participants were on cholesterol lowering medication, three were on antihypertensives, and two were on both. Compliance with the study protocol was very high (>96% as measured by counting capsules in regularly returned containers), and no participants missed more than 3 doses.

One participant dropped out of the study during stage 1 (due to injury), and two withdrew after crossover (one was lost to follow up, another due to developing acne) (Figure 1). All three subjects that withdrew were taking placebo at the time. Thus, data from 45 participants were included into intention-to-treat analyses.

All participants were overweight, most were New Zealand Europeans (89%), and aged 46.5 years (range 34.5–55.6) (Table 2). Their metabolic profiles at baseline are itemized on Table 2. Daily energy intake among participants prior to study is show in Table 2, and was mostly unchanged throughout the trial. There was however, an increased energy intake from sugars during OLE supplementation (17.3 vs 14.7%; p = 0.036). There were no changes in physical activity levels over the study period as assessed by the IPAQ (Placebo = 4651 vs OLE = 4649 METs; p = 0.85).

**Table 2:** Baseline data on the study population (n = 45). Data are mean ± SD, or adjusted means from multivariate models with respective 95% confidence intervals.

**Demographics**	
Age (years)	46.5±5.5
BMI (kg/m^2^)	28.0±2.0
**Diet & Lifestyle**	
Daily energy intake (kcal)	2331±525
Daily energy intake from saturated fat (%)	13.3±3.2
**Glucose homeostasis**	
Insulin sensitivity (Matsuda index)	5.12 (4.31–6.09)
Disposition index	5.17 (2.73–7.74)
**Plasma Lipids**	
Total cholesterol (mmol/l)	5.09 (4.78–5.40)
LDL-C (mmol/l)	3.18 (2.91–3.46)
Oxidised LDL-C (mU/ml)	62552 (57691–67413)
HDL-C (mmol/l)	1.05 (0.97–1.14)
Triglycerides (mmol/l)	1.46 (1.32–1.61)
**Adiposity**	
Total body fat (%)	29.4 (27.7–31.0)
Android fat to gynoid fat ratio	1.31 (1.25–1.37)
**Ambulatory (24-hour) blood pressure**	
Mean diastolic (mmHg)	80.9 (78.7–83.1)
Mean systolic (mmHg)	127.6 (124.4–130.8)
Nocturnal diastolic dipping (%)	18.9 (16.6–21.5)
Nocturnal systolic dipping (%)	13.7 (11.7–15.7)

### Insulin Sensitivity and Other Parameters on Glucose Homeostasis

**Figure 2:**
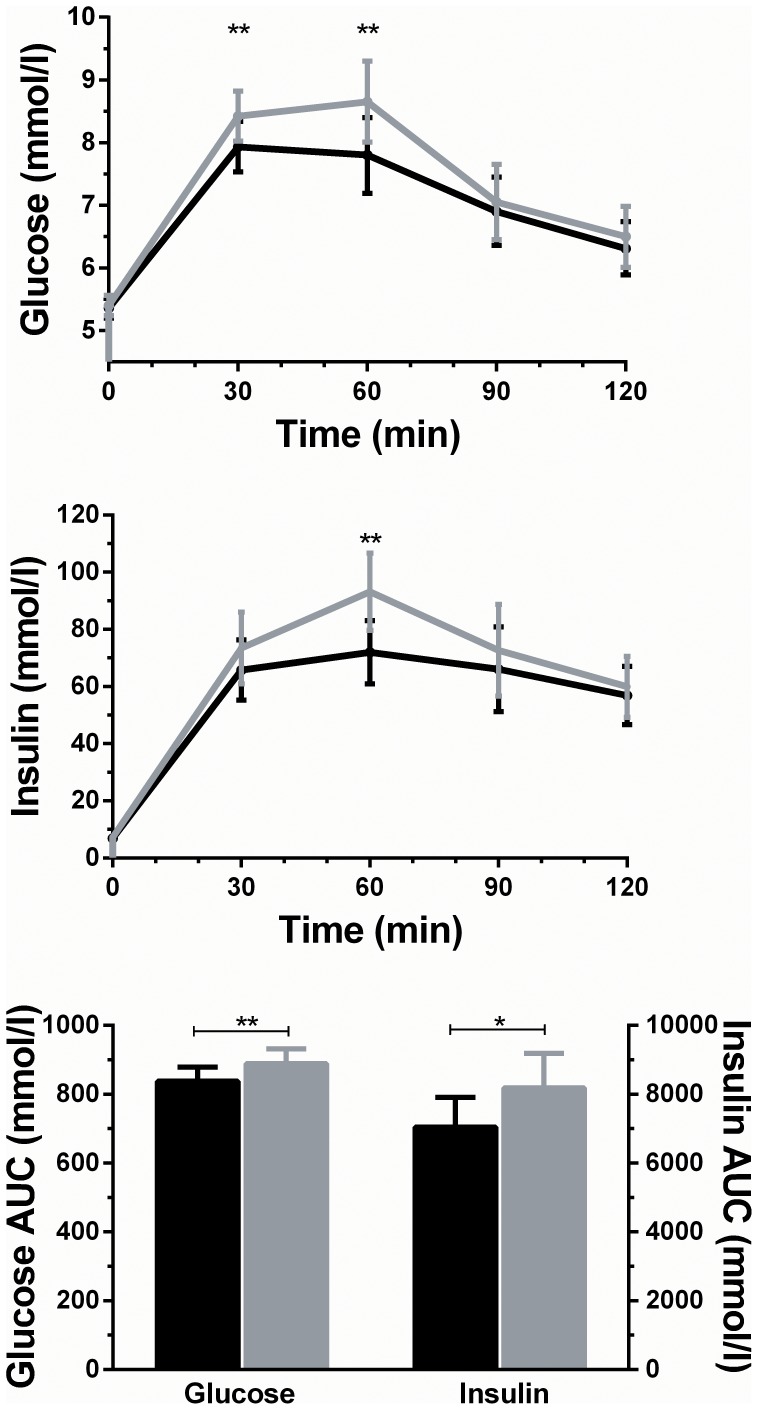
Insulin and glucose responses to oral glucose tolerance tests and respective areas under the curve (AUC), following supplementation with placebo (gray) and olive leaf extract (black). Data are adjusted means from multivariate models with respective 95% confidence intervals.

The assessment of treatment effect (i.e. OLE vs placebo) showed that OLE supplementation was associated with a 15% improvement in insulin sensitivity (5.46 vs 4.73; p = 0.024) (Table 3). Supportive findings included a 28% improvement in pancreatic β-cell function (5.45 vs 4.26; p = 0.013) (Table 3). Further, OLE supplementation also led to a reduction in the area under the curve for both glucose (6%; p = 0.008) and insulin (14%; p = 0.041) (Figure 2). These findings were consistent with observed reductions following OLE treatment in glucose concentrations at 30 (6%; p = 0.008) and 60 (10%; p = 0.005) minutes, as well as a 23% reduction in insulin concentrations at 60 minutes (p = 0.004) (Figure 2).

**Table 3:** Outcomes following a 12-week supplementation with olive leaf extract or placebo (n = 45).

	Placebo	Olive Leaf Extract	p-value
**Glucose homeostasis**			
Insulin sensitivity (Matsuda index)	4.73 (4.13–5.41)	5.46 (4.83–6.16)	**0.024**
Disposition index	4.26 (3.28–5.54)	5.45 (4.14–7.17)	**0.013**
**Hormones**			
IGF-I (ng/ml)	176 (166–186)	181 (172–191)	0.13
IGF-II (ng/ml)	726 (698–754)	7.09 (683–735)	0.14
IGFBP-1 (ng/ml)	1.33 (1.02–1.73)	1.59 (1.28–1.99)	**0.024**
IGFBP-2 (ng/ml)	144 (126–164)	162 (143–183)	**0.015**
IGFBP-3 (ng/ml)	2345 (2203–2507)	2324 (2187–2469)	0.65
**Plasma lipids**			
Total cholesterol (mmol/l)	4.60 (4.39–4.82)	4.72 (4.52–4.94)	0.24
LDL-C (mmol/l)	3.06 (2.87–3.27)	3.10 (2.93–3.28)	0.63
Oxidised LDL-C (mU/ml)	62574 (57378–67770)	62344 (57032–67655)	0.90
HDL-C (mmol/l)	1.07 (1.01–1.13)	1.04 (0.99–1.10)	0.32
Triglycerides (mmol/l)	1.12 (1.01–1.24)	1.16 (1.05–1.29)	0.48
**Proteins**			
Interleukin-6 (pg/ml)	0.57 (0.44–0.75)	0.75 (0.59–0.96)	**0.014**
Interleukin-8 (pg/ml)	1.81 (1.63–1.99)	1.93 (1.72–2.15)	0.11
Ultra-sensitive CRP (ng/ml)	727 (540–978)	702 (543–907)	0.76
TNF-α (pg/ml)	7.57 (7.07–8.10)	7.81 (7.28–8.39)	0.46
**Adiposity**			
Total body fat (%)	30.3 (29.3–30.7)	30.1 (29.3–30.8)	0.89
Android fat to gynoid fat ratio	1.36 (1.33–1.38)	1.36 (1.33–1.38)	1.00
**Ambulatory (24-hour) blood pressure**			
Mean diastolic (mmHg)	78.2 (76.7–79.7)	79.6 (77.8–81.5)	0.088
Mean systolic (mmHg)	126.2 (124.0–128.4)	127.3 (124.8–129.7)	0.33
Nocturnal diastolic dipping (%)	17.6 (15.1–20.0)	17.7 (15.7–19.7)	0.89
Nocturnal systolic dipping (%)	13.6 (11.4–15.7)	13.2 (11.1–15.3)	0.70

Data are adjusted means from multivariate models with respective 95% confidence intervals.

Subjects on OLE also experienced a 32% increase in interleukin-6 (p = 0.014), but there were no observed changes in interleukin-8, TNF-α, or ultra-sensitive CRP (Table 3). While there were no differences in IGF-I, IGF-II, or IGFBP-3 plasma concentrations, OLE supplementation was associated with an increase of 20% in IGFBP-1 (p = 0.024) and 13% in IGFBP-2 (p = 0.015) concentrations (Table 3). There were no significant changes in lipid profile (including oxidised LDL-C), ambulatory blood pressure, body composition (Table 3), or carotid intima-media thickness (OLE 0.820 (0.782–0.859) vs Placebo 0.832 (0.795–0.871) mm; p = 0.40). There were also no significant changes in subjective assessment of wellbeing (data not shown).

### Adverse Outcomes

The only adverse event reported was a flare up of acne. The participant withdrew from the study and un-blinding showed that he was receiving placebo. Liver function tests showed no differences in AST, ALP, ALT, or GGT among participants in OLE vs placebo (data not shown).

### Subgroup Analyses

Data were also analysed on a subgroup of 36 participants, excluding 9 subjects who were on lipid-lowering and/or anti-hypertensive medications (Table 4). The results changed very little, but importantly, there was evidence of an even greater effect of OLE on insulin sensitivity (20%) compared to placebo (5.94 vs 4.96; p = 0.009) (Table 4).

**Table 4:** Outcomes following a 12-week supplementation with olive leaf extract or placebo.

	Placebo	Olive Leaf Extract	p-value
**Glucose homeostasis**			
Insulin sensitivity (Matsuda index)	4.96 (4.35–5.65)	5.94 (5.32–6.62)	**0.009**
Disposition index	4.87 (3.80–6.25)	6.03 (4.59–7.93)	**0.050**
**Hormones**			
IGF-I (ng/ml)	175 (165–185)	178 (169–188)	0.43
IGF-II (ng/ml)	701 (670–732)	689 (670–708)	0.37
IGFBP-1 (ng/ml)	1.75 (1.38–2.11)	2.28 (1.78–2.77)	**0.004**
IGFBP-2 (ng/ml)	166 (149–185)	194 (175–216)	**0.003**
IGFBP-3 (ng/ml)	2317 (2200–2439)	2324 (2197–2458)	0.90
**Plasma lipids**			
Total cholesterol (mmol/l)	4.78 (4.60–4.98)	4.84 (4.64–5.06)	0.63
LDL-C (mmol/l)	3.28 (3.13–3.44)	3.30 (3.13–3.47)	0.89
Oxidised LDL-C (mU/ml)	66927 (62918–70936)	67433 (61965–72902)	0.82
HDL-C (mmol/l)	1.08 (1.03–1.14)	1.04 (0.99–1.10)	0.15
**Proteins**			
Interleukin-6 (pg/ml)	0.49 (0.37–0.63)	0.64 (0.53–0.78)	**0.028**
Interleukin-8 (pg/ml)	1.71 (1.58–1.84)	1.92 (1.74–2.12)	**0.008**
Ultra-sensitive CRP (ng/ml)	793 (640–983)	586 (711–1037)	0.45
TNF-α (pg/ml)	7.42 (7.01–7.84)	7.86 (7.32–8.43)	0.19
**Adiposity**			
Total body fat (%)	29.9 (29.3–30.6)	30.1 (29.4–30.9)	0.60
Android fat to gynoid fat ratio	1.33 (1.30–1.36)	1.33 (1.30–1.35)	0.72
**Ambulatory (24-hour) blood pressure**			
Mean diastolic (mmHg)	75.9 (74.6–7.3)	77.2 (75.3–79.2)	0.17
Mean systolic (mmHg)	122.7 (120.7–124.7)	123.9 (121.4–126.5)	0.34
Nocturnal diastolic dipping (%)	16.8 (14.5–19.1)	18.0 (15.8–20.1)	0.42
Nocturnal systolic dipping (%)	12.8 (10.8–14.7)	12.6 (10.3–14.9)	0.88

Analyses excluded 9 participants on lipid-lowering and/or antihypertensive medications, so that n = 36. Data are adjusted means from multivariate models with respective 95% confidence intervals.

## Discussion

We have shown that supplementation with olive leaf polyphenols for 12 weeks improves two aspects of glucose regulation (both insulin action and secretion) in a cohort of overweight middle-aged men. This novel finding was independent of lifestyle factors (such as dietary intakes and physical activity levels), BMI, or fat distribution. Importantly, the 15–20% improvement in insulin sensitivity observed with OLE supplementation is comparable to those seen with medications commonly used to treat diabetes. For example, metformin (250 mg TDS) improved insulin sensitivity by 17% in a group of sedentary overweight non-diabetics [21]. However, as Ou et al.’s cohort reported lower levels of physical activity than our participants [21], the use of metformin in our study group would likely have led to a comparatively smaller improvement in insulin sensitivity. Thus, we speculate that the observed improvement in insulin sensitivity with OLE is greater than would have otherwise been observed if our subjects have been treated with metformin instead. Another study demonstrated a 28% improvement in insulin sensitivity after treatment with 30 mg pioglitazone for 26 weeks [22]; but as their participants had type 2 diabetes, they are also likely to have shown an exaggerated response compared to our study group.

In addition, OLE also improved insulin secretion to further aid glucose regulation, which does not occur with the use of metformin. Type 2 diabetes generally involves defects in both insulin sensitivity and pancreatic β-cell secretory capacity [23], [24]. OLE supplementation was associated with a reduction in the glucose and insulin excursion after oral glucose challenge, suggesting an improvement in both pancreatic β-cell function and insulin sensitivity. The observed 28% improvement in disposition index is consistent with this observation. Comparatively, studies in diabetic adults (who are likely to have an exaggerated response to therapy) have shown that mainstream medications affecting only β-cell secretion capacity have achieved improvements of 55% (dipeptidyl peptidase-4 antagonists) [25] and 100% (glucagon-like peptide-1 agonists) [26]. Hence, compared to these drugs that only improve insulin secretion, OLE improves both insulin sensitivity and pancreatic β-cell secretory capacity. Remarkably, the observed effects of OLE supplementation in our study population is comparable to common diabetic therapeutics (particularly metformin), and our results could have clinical significance for patients with type 2 diabetes.

Only one randomized placebo-controlled trial has previously investigated the effects of OLE on glucose metabolism in subjects with type 2 diabetes, finding an improvement in glycated haemoglobin (HbA1c) after 14 weeks of supplementation [27]. However, that study did not measure or discuss possible variations in diet or levels of physical activity among participants [27], so that the independent effect of OLE cannot be determined. Hence, our study is the first to show the independent effects of OLE on glucose homeostasis in humans, corroborating previous findings *in vitro* and in animal models [5].

We also found elevated interleukin-6 levels (a pro-inflammatory cytokine) with OLE supplementation. Interleukin-6 functions differently depending on its concentration and the tissue it acts upon. Acute increases improve the insulin-regulated glucose metabolism in the muscle [28], while chronically mildly elevated levels are associated with a pro-inflammatory insulin resistant state in the liver. Thus, OLE supplementation may improve insulin sensitivity and glucose uptake via interleukin-6, and possible mechanisms for this effect have been proposed [29], [30]. Further, we also observed that OLE supplementation led to increased IGFBP-1 and IGFBP-2 plasma concentrations. Increased IGFBP-2 concentrations are protective against the development of obesity and improve insulin sensitivity [31], while higher IGFBP-1 concentrations are associated with lower insulin levels [32].

In regards to other measured cardiovascular outcomes, OLE supplementation did not improve 24-hour ambulatory blood pressure, lipid profile, or cIMT. Previous studies have shown improvements in blood pressure with OLE supplementation [33], [34], but they did not involve 24-hour monitoring. Similarly, our findings on lipid profile also contrast with those of previous studies [33], [34], [35]. However, Perrinjaquet-Moccetti et al. did not examine dietary factors [33], Susalit et al. had a low cholesterol dietary component to the trial [34], and Fonolla et al. studied hypercholesterolemic subjects [35]. In addition, although we did not observe improvements in cIMT, this null result may be a result of our relatively short intervention. Nonetheless, consistent with our findings, the European Food Safety Authority recently concluded that there was insufficient evidence to substantiate health claims of improvements on blood pressure, lipid profile, or anti-inflammatory effects [8].

The strengths of this study lie with it being a randomized, double-blinded, placebo-controlled, crossover trial, using well-validated scientific methods (i.e. ambulatory blood pressure, Matsuda method, and cIMT). Although insulin sensitivity was not measured using the gold-standard euglycemic hyperinsulinemic clamp, it was assessed using the Matsuda method that is one of the best performing proxy methods [11]. In addition, we adopted a comprehensive approach to modifiable cardiovascular risk factors, including attention to dietary intakes and physical activity levels. The OLE supplement was well-tolerated, and compliance with the study protocol was excellent. Potential weaknesses include the relatively short intervention, which may have obscured pathophysiological changes that require longer periods of time to develop.

Overall, this is the largest and most comprehensive study to date examining the effect of supplemented olive leaf polyphenols alone on modifiable cardiovascular risk factors. We showed improvements in insulin sensitivity and pancreatic β-cell secretion capacity, in a cohort of overweight middle-aged men. Future research should evaluate the potential effects of olive leaf polyphenols on insulin sensitivity and glycaemic control (HbA1c) in patients with type 2 diabetes, and compare any such effects to conventional therapy (e.g. metformin).

## Supporting Information

Protocol S1 **Trial Protocol.**
(DOCX)

Checklist S1 **CONSORT Checklist.**
(DOC)
