# Supplementary material for: Olive (Olea europaea L.) Leaf Polyphenols Improve Insulin Sensitivity in Middle-Aged Overweight Men: A Randomized, Placebo-Controlled, Crossover Trial
Source: PLoS One. 2013 Mar 13;8(3):e57622. doi: 10.1371/journal.pone.0057622 (PMC3596374; doi:10.1371/journal.pone.0057622)
Supplement: Protocol S1 — Trial Protocol. (DOCX) [file pone.0057622.s001.docx]

**Protocol S1 – Trial protocol**

***Trial registration:*** *ACTRN#336317 (Australian New Zealand Clinical Trials Registry).*

A 30-week randomized, placebo-controlled, crossover trial of 12 weeks of either **olive polyphenols** in the form olive leaf extract (OLE) or **placebo**, in middle-aged overweight men. After 12 weeks, subjects supplemented with olive polyphenols will cross over to placebo, and the subjects on placebo will crossover to receive olive polyphenols. There is a six week washout between phases. Olive polyphenols will be in the form of 4 capsules of olive leaf extract, delivery 51.1 mg of oleuropein, and 9.7 mg of hydroxytyrosol. All subjects will be formally assessed at 3 time points; 1) baseline, 2) crossover (at 12 weeks), and 3) study completion (30 weeks). Each assessment will be performed at the Liggins Institute (Maurice and Nessie Paykel Clinical Research Unit). Participants will be recruited by newspaper advertising. Participant randomization will be carried by computer random number generation, with participants and investigators blinded to allocation until after statistical analysis.

**Inclusion criteria**

1. Males
2. Age 35–55 years
3. BMI 25–30 kg/m^2^

**Exclusion Criteria**

1. Receiving medications that influence insulin sensitivity (including oral steroids and metformin)
2. Smokers
3. Diabetes

12 weeks

12 weeks

Assessment

Assessment

Assessment

**Assessments**

Prior to arrival for the assessments, all participants will be instructed to fast for 8 hours, and avoid strenuous exercise for the preceding 24 hours. On arrival, resting energy expenditure will be assessed first before IV insertion. All blood samples will be centrifuged, separated into plasma and serum, and frozen at -20°C for later analysis. Following each assessment, subjects will be offered a meal.

**Primary outcome**

Insulin sensitivity as determined by the Matsuda method. Subjects will be fasted overnight. Upon arrival an IV cannula will be inserted, and fasting blood sample taken. The Matsuda method uses a 1.75 g/kg (max 75 g) glucodex oral glucose tolerance challenge with plasma glucose and insulin levels taken at 0, 30, 60, 90, and 120 min. Matsuda index is highly correlated with clamp-derived insulin sensitivity indices.

**Secondary outcomes**

1. Body weight (to 0.1 kg), height (wall-mounted stadiometer to 0.1cm). BMI calculated as weight (kg) / height (m)^2^.
2. Body composition and bone mineral density (DEXA – Lunar Prodigy).
3. 24-hour ambulatory blood pressure, using Spacelabs ambulatory blood pressure monitoring system. This will occur within 7 days before the assessments.
4. Wellness questionnaire – using the SF36 online tool. Participants will logon with unique code during the assessment.
5. Hand grip strength – measured with a Jamar dynamometer. Each subject will stand, with elbow slightly flexed, and exert maximum pressure. The average of 3 attempts will be recorded as per the manufacturer instructions.
6. Resting energy expenditure – determined by indirect calorimetry using a TrueOne 2400 Metabolic Measurement system (Parvo Medics Inc., Yorba Sandy, Utah). Subjects will lie supine in a quiet temperature-controlled environment. The first 10 minutes will be discarded, then 30-second energy expenditure values from 10–30 minutes will be recorded, and an average value calculated to give a final score.
7. Carotid intima thickness measurement – measured on the far wall of the right carotid artery using carotid ultrasound (M-Turbo), 10 mm from the carotid bulb. The largest measurement from at least 5 images taken will be recorded.
8. Insulin sensitivity biomarkers – fasting blood samples will be used to measure serum IGF-I, IGF-II, IGFBP-1, IGFBP-3 IL-6, TNFα, CRP. ELISA kits will be used.
9. Lipid profile – fasting blood samples for plasma total cholesterol, triglycerides, LDL-C and HDL-C.
10. Liver function tests – fasting blood samples for GGT, ALT, AST, ALP.
11. Oxidized LDL – fasting sample.
12. Bone turnover – fasting blood sample for serum bone-specific ALP (ELISA).
13. Physical activity – IPAQ questionnaire recording physical activity for the prior 7 days will occur at each assessment.
14. Dietary intake – 3-day food records (2 work days, one weekend) will be collected at each assessment. Energy and macronutrient intakes calculated using FoodWorks Software.
15. Side effect monitoring – at each assessment subjects will be asked to report any side effects from the intervention.

Following statistical analysis, all subjects will be informed of their individual results. Any concerning results will be discussed with them and referred to their General Practitioner.
